# Follistatin-based ligand trap ACE-083 induces localized hypertrophy of skeletal muscle with functional improvement in models of neuromuscular disease

**DOI:** 10.1038/s41598-019-47818-w

**Published:** 2019-08-06

**Authors:** R. S. Pearsall, M. V. Davies, M. Cannell, J. Li, J. Widrick, A. W. Mulivor, S. Wallner, M. E. Troy, M. Spaits, K. Liharska, D. Sako, R. Castonguay, S. Keates, A. V. Grinberg, R. N. V. S. Suragani, R. Kumar

**Affiliations:** 1 Acceleron Pharma, Cambridge, MA USA; 2 Division of Genetics and Genomics, The Manton Center for Orphan Disease Research, Boston Children’s Hospital, Harvard Medical School, Boston, MA USA; 3 Present Address: The Hospital for Sick Children, Toronto, Ontario Canada; 4 Present Address: NovaRock Biotherapeutics, Princeton, NJ USA; 5 Present Address: Dragonfly Therapeutics, Waltham, MA USA

**Keywords:** Drug development, Pharmacodynamics

## Abstract

Skeletal muscle is under inhibitory homeostatic regulation by multiple ligands of the transforming growth factor-β (TGFβ) superfamily. Follistatin is a secreted protein that promotes muscle growth and function by sequestering these ligands extracellularly. In the present study, we evaluated the potential of ACE-083 – a locally acting, follistatin-based fusion protein – as a novel therapeutic agent for focal or asymmetric myopathies. Characterization of ACE-083 *in vitro* revealed its high affinity for heparin and extracellular matrix while surface plasmon resonance and cell-based assays confirmed that ACE-083 binds and potently neutralizes myostatin, activin A, activin B and growth differentiation factor 11 (GDF11). Intramuscular administration of ACE-083 caused localized, dose-dependent hypertrophy of the injected muscle in wild-type mice and mouse models of Charcot-Marie-Tooth disease (CMT) and Duchenne muscular dystrophy, with no evidence of systemic muscle effects or endocrine perturbation. Importantly, ACE-083 also increased the force of isometric contraction *in situ* by the injected tibialis anterior muscle in wild-type mice and disease models and increased ankle dorsiflexion torque in CMT mice. Our results demonstrate the potential of ACE-083 as a therapeutic agent for patients with CMT, muscular dystrophy and other disorders with focal or asymmetric muscle atrophy or weakness.

## Introduction

Weakness or loss of skeletal muscle can arise from multiple causes, including hereditary neuromuscular disorders, acquired diseases, sarcopenia, trauma, athletic injuries and disuse. A frequent characteristic of muscle disorders is focal or asymmetric loss of function^1^. One example of focal muscle loss is Charcot-Marie-Tooth disease (CMT), in which distal leg weakness and foot drop typically lead to reduced mobility and increased risk of falls and injury^2^. Among muscular dystrophies is facioscapulohumeral muscular dystrophy (FSHD), which is characterized clinically by progressive muscle weakening in a focal and asymmetric manner typically involving facial, scapular, upper arm, lower leg, and abdominal muscles^3^. Patients with these and related disorders would likely benefit from therapeutic agents providing focal improvement in muscle function.

Skeletal muscle is under inhibitory homeostatic regulation by multiple ligands that activate the Smad2/3 branch of the TGFβ superfamily signaling pathway^4^. Myostatin (GDF8) is a well-established endogenous inhibitor of myogenesis^5^. In addition, activin A and activin B are implicated as endogenous inhibitors of muscle growth that act synergistically with myostatin^6–9^. GDF11, which is structurally similar to myostatin, can also inhibit muscle regeneration or induce muscle atrophy under certain conditions^10–12^. The relative contribution of these individual ligands to inhibition of muscle mass differs between rodents and primates under basal conditions^9^ and may vary with disease state^8,13^. For these reasons, concurrent inhibition of multiple TGFβ superfamily ligands is likely to be a more promising approach to maximize muscle growth and associated functional improvement than inhibition of any single ligand.

Follistatin is a naturally-occurring protein that acts as an extracellular trap to regulate biologic activity of the aforementioned TGFβ superfamily ligands. Follistatin isoforms each contain a heparin-binding site (HBS) but differ in the extent to which their C-terminal domain masks this HBS, thereby altering affinity of follistatin for cell-surface glycoproteins and extracellular matrix. Thus, the longest isoform (FST315) is considered to be the major circulating form of follistatin, whereas the shortest (FST288) is thought to act locally due to its high affinity for heparan sulfate^14,15^. Therapeutic interventions based on these follistatin isoforms, or derivatives thereof, have demonstrated efficacy in normal animals and disease models through improvements in muscle mass, strength, regeneration and fibrosis^16–22^. These studies provide a compelling rationale for follistatin-based therapies as systemically acting inducers of muscle growth but do not address the potential of such agents for focused local therapy.

We recently demonstrated the feasibility of using a FST288-based fusion protein to promote dose-dependent, localized growth of skeletal muscle in normal mice without detectable systemic effects^23^. The objective of the present study was to evaluate ACE-083 – a novel, FST291-Fc fusion protein currently in clinical development^24–26^ – for its ability to produce focal muscle hypertrophy and increased strength in normal mice and murine models of neuromuscular disease.

## Results

### Structure and binding properties of ACE-083 *in vitro*

**Figure 1:**
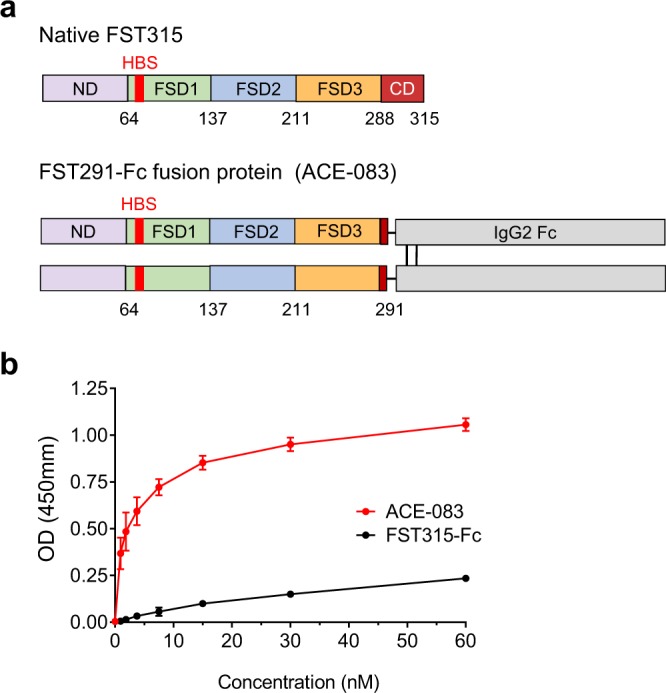
Follistatin-based fusion protein ACE-083 displays high affinity for extracellular matrix. (**a**) ACE-083 differs from native FST315 by nearly complete removal of the C-terminal domain (CD) and by attachment to an immunoglobulin Fc domain, which results in dimerization of the follistatin polypeptide. ND, N-terminal domain; FSD, follistatin domains; HBS, heparin-binding site. (**b**) Binding of ACE-083 or FST315-Fc to extract of extracellular matrix (Matrigel) as detected by colorimetric assay. Data are means ± SD of three separate experiments each performed in triplicate.

ACE-083 is a novel, dimeric fusion protein in which human FST291 is coupled to the Fc domain of human IgG2 (Fig. 1a) to obtain pharmacokinetic properties similar to those of an antibody^27^. Additionally, attachment of an Fc domain simplifies protein purification and doubles the number of heparin binding sites (HBS) per molecule as a consequence of dimerization.

We hypothesized that ACE-083 would possess high affinity for heparin and heparan-sulfate proteoglycans – and thus higher retention in muscle tissue – due to nearly full truncation of the C-terminal domain in the FST315 portion of the fusion protein. In a preliminary assessment of heparin-binding affinity, we measured the concentration of sodium chloride required to displace bound ACE-083, or a corresponding fusion protein containing full-length FST315 (FST315-Fc), from an analytical heparin HPLC column. Elution of ACE-083 from the column required a higher concentration of sodium chloride (938 mM) than did FST315-Fc (792 mM), indicative of ACE-083 having greater affinity for heparin. To confirm this result under conditions more closely resembling an extracellular environment, ACE-083 and FST315-Fc were evaluated for their binding to Matrigel®, a commercially available extract of extracellular matrix^28^. In this semi-quantitative assay, ACE-083 displayed a concentration-dependent affinity for Matrigel® markedly exceeding that of FST315-Fc (Fig. 1b). These data indicate that ACE-083 has high affinity for heparin and proteins derived from extracellular matrix.

We next used surface plasmon resonance (SPR) biosensor methodology to characterize direct binding of ACE-083 to TGFβ superfamily ligands that negatively regulate skeletal muscle growth. ACE-083 was captured on an anti-human IgG Fc sensor chip, and different concentrations of activin A, activin B, myostatin or GDF11 were injected over the captured ACE-083 as analytes. The resulting sensorgrams are shown online in Supplementary Fig. S1 while a summary of parameters for ligand binding to ACE-083 is presented in Table 1. For these four ligands, the apparent equilibrium dissociation constant (*K*_D_) ranged from approximately 10–350 pM, and the dissociation rate constant (*k*_d_) ranged from approximately 7 × 10^−5^ to 1.5 × 10^−4^ s^−1^. These data confirm that ACE-083 binds tightly to key ligands (myostatin, activin A and activin B) involved in the inhibitory homeostatic regulation of skeletal muscle mass as well as GDF11, whose role in the local muscle environment remains unclear. The slow dissociation rate of these ligands from ACE-083 further confirms effectiveness of ACE-083 as a ligand trap under cell-free conditions.

**Table 1:** Ligand-binding parameters and inhibitory potency for ACE-083.

Ligand	Surface Plasmon Resonance			Reporter-Gene Assay
	*k*_a_ (M^−1^ s^−1^)	*k*_d_ (s^−1^)	*K*_D_ (pM)	IC_50_ (pM)
Activin A	1.97 × 10^6^	1.55 × 10^−4^	78.8	39.2
Activin B	1.93 × 10^7^	1.99 × 10^−4^	10.3	49.2
GDF8	5.32 × 10^5^	1.85 × 10^−4^	348	676
GDF11	4.77 × 10^6^	7.20 × 10^−5^	15.4	75.9

ACE-083 binding to TGFβ superfamily ligands as determined by surface plasmon resonance and inhibitory potency as determined by cell-based reporter-gene assay. Data for kinetic characterization were duplicates globally fit to a 1:1 binding model with mass transfer term using BIAevaluation software. SPR assays were carried out for ACE-083 more than three times, and these data represent means of two experiments performed on separate flow cells. Inhibitory activity of ACE-083 was measured in dual-luciferase reporter assays conducted with A204 cells. IC_50_ values were calculated with GraphPad Prism and are the means of three independent assays, each containing duplicate samples.

Follistatin binds several BMPs, including BMP4, BMP6, BMP7, and BMP15, in certain contexts^29,30^. Therefore, we used surface plasmon resonance to screen BMPs for their ability to interact with ACE-083 under cell-free conditions. Among BMPs, only BMP6 and BMP7 exhibited binding to ACE-083 (see Supplementary Table 1).

We then investigated whether ACE-083 is able to neutralize signaling by activins, myostatin, and GDF11 through their cognate receptors in a cellular environment. In a reporter-gene assay conducted with a human rhabdomyosarcoma (A204) cell line, ACE-083 inhibited signaling by activin A, activin B, myostatin and GDF11 in a dose-dependent manner, with IC_50_ values ranging from approximately 40–700 pM (Table 1) or, in alternative units, 5–80 ng/mL (see Supplementary Fig. S2). We also confirmed with a separate reporter-gene assay in T98G cells that ACE-083 at concentrations up to 20 µg/mL does not inhibit signaling by the vascular regulatory ligand BMP9^31^. These data demonstrate that ACE-083 can potently inhibit signaling by activin A, activin B, myostatin and GDF11 in a cellular environment.

### Focal activity of ACE-083 in skeletal muscle of wild-type mice

**Figure 2:**
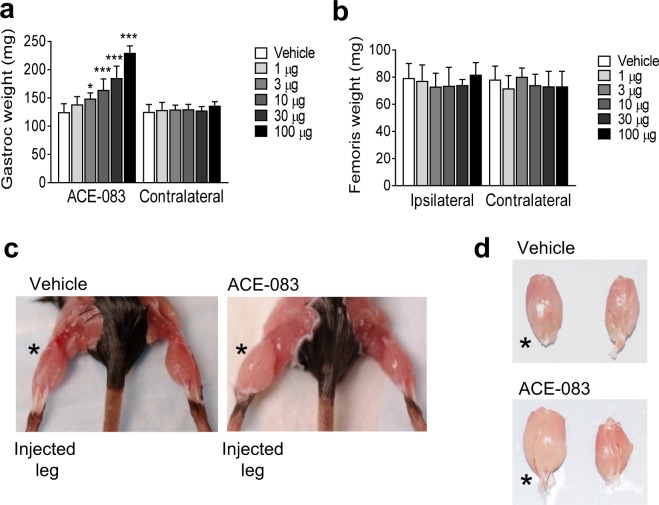
Local administration of ACE-083 in wild-type mice causes dose-dependent growth of the injected skeletal muscle. The gastrocnemius muscle received unilateral injections of ACE-083 or vehicle (phosphate-buffered saline, PBS) twice weekly for 4 weeks. (**a**) Weight of injected gastrocnemius muscle after ACE-083 treatment compared to uninjected, contralateral gastrocnemius. Data are means ± SEM (n = 8 per group). *P ≤ 0.05, ***P ≤ 0.001 vs. vehicle by one-way ANOVA with Dunnett’s adjustment. (**b**) Weight of uninjected rectus femoris muscle (ipsilateral to injected gastrocnemius muscle) and uninjected, contralateral rectus femoris muscle. Data are means ± SEM (n = 8). (**c**) Representative images of wild-type mice treated i.m. with vehicle or ACE-083 (100 µg). (**d**) Representative images of excised gastrocnemius muscles from wild-type mice treated i.m. with vehicle or ACE-083 (100 µg). * indicates injected gastrocnemius.

In the first of a series of experiments in mice, we investigated whether intramuscular (i.m.) administration of ACE-083 in wild-type mice causes growth of the injected muscle and potentially exerts systemic effects. Unilateral i.m. administration of ACE-083 in the gastrocnemius muscle of C57BL/6J mice (Jax #000664) twice weekly for 4 weeks increased the weight of the injected muscle in a dose-dependent manner (Fig. 2a). The highest dose of ACE-083 tested (100 μg) increased muscle mass by 85% compared to the corresponding gastrocnemius muscle in vehicle-treated mice (Fig. 2a,c,d). None of the ACE-083 doses tested altered weights of nontargeted muscles, including the contralateral gastrocnemius (Fig. 2a,c,d), adjacent (ipsilateral) rectus femoris (Fig. 2b) or contralateral rectus femoris (Fig. 2b). Representative images illustrate the selective effect of ACE-083 administration on the injected gastrocnemius compared to vehicle injection or uninjected, contralateral gastrocnemius muscles (Fig. 2c,d). Unilateral i.m. administration of ACE-083 did not appreciably alter body weight or circulating levels of follicle-stimulating hormone (see Supplementary Fig. S3), whose secretion is inhibited by endogenous follistatin^32^. These data indicate that locally administered ACE-083 causes focal growth of skeletal muscle in wild-type mice without evidence of systemic effects.

**Figure 3:**
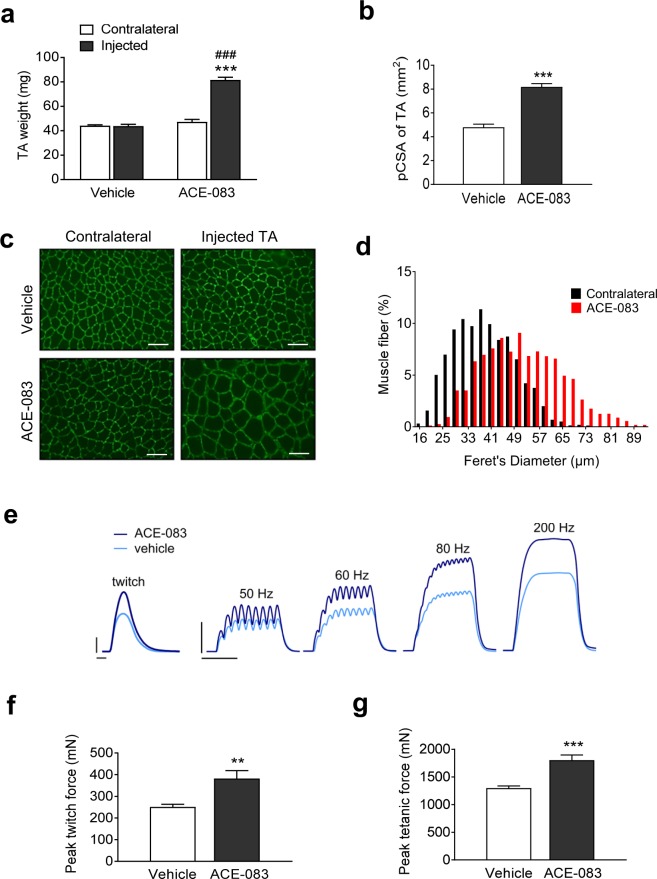
Local administration of ACE-083 in wild-type mice causes localized hypertrophy of skeletal muscle accompanied by increased force generation. Mice were given unilateral injections of ACE-083 (100 µg) or vehicle (PBS) in the tibialis anterior (TA) muscle twice weekly for 4 weeks. (**a**) Weights of ACE-083-injected, vehicle-injected and uninjected (contralateral) TA muscles. Data are means ± SEM (n = 8–9 per group). ***P ≤ 0.001 vs. vehicle by unpaired t-test; ^###^P ≤ 0.001 vs. contralateral TA by one-way ANOVA with Tukey’s adjustment. (**b**) Physiological cross-sectional area (pCSA) of ACE-083-injected and vehicle-injected TA muscles. Data are means ± SEM (n = 8–9). ***P ≤ 0.001 vs. vehicle by unpaired t-test. (**c**) Photomicrographs of representative cross-sections through ACE-083-injected, vehicle-injected, and uninjected (contralateral) TA muscles in which an antibody directed against laminin was used to delineate individual fibers. Scale bar, 100 µm. (**d**) Histograms of muscle fiber diameter in ACE-083-injected and uninjected (contralateral) TA muscles. Data represent 200 fibers per muscle (n = 8 mice). (**e**) Representative force responses to a single stimulus (twitch) and increasing stimulation frequency for ACE-083-injected and vehicle-injected TA muscles. Calibration bars: twitch, 100 mN and 20 ms; 50–200 Hz, 500 mN and 100 ms. **(f**) Peak twitch force generated by ACE-083-injected and vehicle-injected TA muscles as determined *in situ*. Data are means ± SEM (n = 8–9). **P ≤ 0.01 vs. vehicle by unpaired t-test. (**g**) Peak tetanic force generated by ACE-083-injected and vehicle-injected TA muscles as determined *in situ*. Data are means ± SEM (n = 8–9). ***P ≤ 0.001 vs. vehicle by unpaired t-test.

We next sought to determine whether increased muscle weight caused by local administration of ACE-083 reflects muscle fiber hypertrophy. We focused on the tibialis anterior (TA) muscle in these experiments because its weakness in certain diseases impairs ankle dorsiflexion and thereby restricts patient mobility^2^. Unilateral i.m. administration of ACE-083 (100 µg) in the TA muscle of wild-type (C57BL/6J) mice twice weekly for 4 weeks increased the weight of the injected muscle by approximately 75% compared to vehicle (PBS) or the contralateral, uninjected TA (Fig. 3a). The physiological cross-sectional area (pCSA) – as determined by the formula pCSA = M/(FL × D), where M is muscle mass, FL is fiber length and D is muscle density – was also increased in the ACE-083-injected muscle compared to vehicle-injected muscle (Fig. 3b). To determine whether this ACE-083-generated increase in cross-sectional area was a result of muscle fiber hypertrophy rather than hyperplasia, cross sections through the TA were labeled with an antibody against the sarcolemmal protein laminin to delineate individual muscle fibers. Fibers in the ACE-083-injected muscle were visibly enlarged compared with the vehicle-injected or contralateral muscles (Fig. 3c). Quantitative analysis of muscle fiber diameters in ACE-083-injected and uninjected (contralateral) TA muscles from all mice treated with ACE-083 revealed a pronounced rightward shift in the frequency distribution of fiber diameter as a function of ACE-083 treatment (Fig. 3d). For example, 55% of fibers from ACE-083-injected TA muscles were at least 50 μm in diameter, whereas only 18% of fibers from contralateral, uninjected TA muscles were that size (Fig. 3d). These data demonstrate that local administration of ACE-083 in wild-type mice causes focal muscle growth due to fiber hypertrophy. As determined by Western blot, ACE-083 caused a decrease in phosphorylated Smad3 and an increase in phosphorylated Akt (see Supplementary Figs S4 and S5 and Methods). These signaling pathways are known to promote muscle growth, and the changes are consistent with what has been previously described for follistatin^8,33^.

We next investigated whether the muscle fiber hypertrophy caused by local administration of ACE-083 is accompanied by increased isometric force generation. Wild-type mice were given unilateral injections of ACE-083 (100 µg) in the TA muscle twice weekly for 4 weeks. Figure 3e shows representative force responses by ACE-083- and vehicle-injected TA muscles to a single supramaximal pulse (0 Hz, twitch response) and to pulse trains of increasing frequency (submaximal and maximal responses). ACE-083-injected muscles generated greater absolute force than vehicle-treated muscles at all stimulation frequencies (Fig. 3e). Group data indicate that the absolute twitch force generated by ACE-083-treated TA muscles was 52% greater than that of vehicle-treated muscles (Fig. 3f), while the absolute peak tetanic force generated by ACE-083-treated TA muscles was 40% greater than that of vehicle-treated counterparts (Fig. 3g). When the change in absolute force was normalized to the increase in pCSA, there was no significant difference between vehicle and ACE-083 treatment (see Supplementary Fig. S6). These results indicate that muscle fiber hypertrophy caused by local administration of ACE-083 in wild-type mice increases absolute but not specific force, consistent with previous assessments of altered Smad2/3 pathway signaling due to systemic interventions^34^.

### Focal skeletal muscle activity of ACE-083 in a mouse model of Charcot-Marie-Tooth (CMT) disease

**Figure 4:**
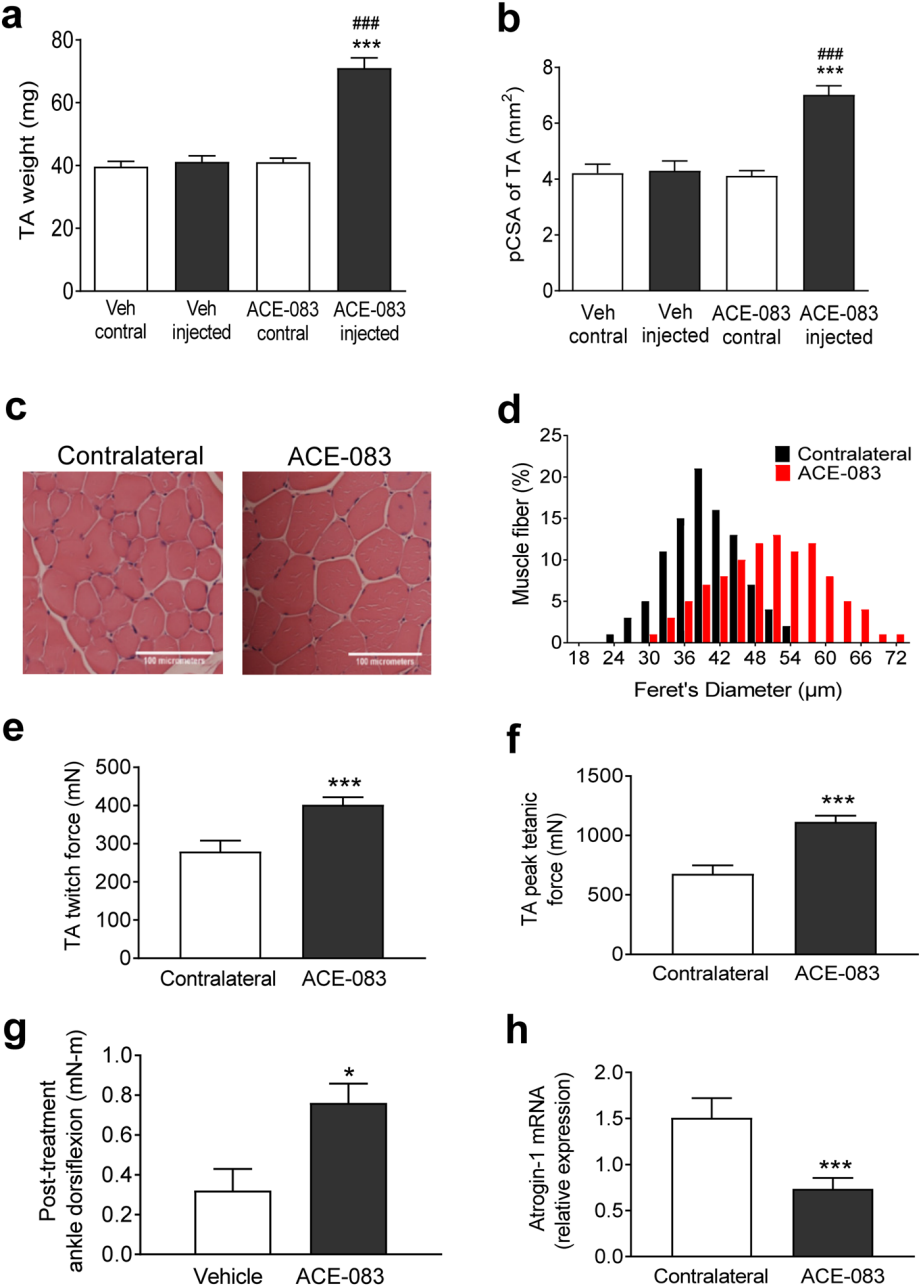
Local administration of ACE-083 in a Trembler^J^ mouse model of CMT causes focal hypertrophy of skeletal muscle, increases muscle force generation and improves ankle dorsiflexion torque. TrJ/Pmp22 mice (CMT mice) were given unilateral injections of ACE-083 (100 µg) or vehicle (PBS) in the TA muscle twice weekly for 4 weeks. (**a**) Weights of ACE-083-injected, vehicle-injected, and uninjected (contralateral) TA muscles. Data are means ± SEM (n = 10). ***P ≤ 0.001 vs. vehicle; ^###^P ≤ 0.001 vs. uninjected (contralateral) TA by one-way ANOVA with Tukey’s adjustment. (n = 10–12). (**b**) Physiological cross-sectional area of ACE-083-injected, vehicle-injected, and uninjected (contralateral) TA muscles. Data are means ± SEM (n = 7–11). ***P ≤ 0.001 vs. vehicle; ^###^P ≤ 0.001 vs. uninjected (contralateral) TA by one-way ANOVA with Tukey’s adjustment. (n = 7–11). (**c**) Photomicrographs of representative cross-sections stained with haematoxylin-eosin to demarcate individual fibers from ACE-083-injected and uninjected (contralateral) TA muscles. Scale bar, 100 µm. (**d**) Histograms of muscle fiber diameter in ACE-083-injected and uninjected (contralateral) TA muscles. Data represent 200 fibers per muscle (n = 10 mice). (**e**) Peak twitch force generated by ACE-083-injected or uninjected (contralateral) TA muscles as determined *in situ*. Data are means ± SEM (n = 5). ***P ≤ 0.001 vs. contralateral TA by unpaired t-test. (**f**) Peak tetanic force generated by ACE-083-injected or uninjected (contralateral) TA muscles as determined *in situ*. Data are means ± SEM (n = 5). ***P ≤ 0.001 vs. contralateral TA by unpaired t-test. (**g**) Ankle dorsiflexion torque determined *in vivo* under isometric conditions in ACE-083-injected or vehicle-injected CMT mice. Data are means ± SEM (n = 5). *P ≤ 0.05 vs. vehicle-injected mice. (**h**) Levels of *Fbxo32* mRNA encoding muscle atrophy biomarker atrogin-1 in ACE-083-injected or uninjected (contralateral) TA muscles. Data are means ± SEM (n = 11). **P ≤ 0.001 vs. uninjected (contralateral) TA by Mann-Whitney *U* test.

We next evaluated the ability of ACE-083 to induce focal muscle hypertrophy in the Trembler-J mouse model of CMT1A^35^, which harbors a mutation in peripheral myelin protein 22 (*Pmp22*), a gene critical in the etiology of CMT1A. Seven-month-old B6.D2-Pmp22Tr-J/J mice (CMT mice) were given unilateral injections of ACE-083 (100 µg) or vehicle (PBS) in the TA muscle twice weekly for 4 weeks. ACE-083 treatment increased TA muscle weight by 73% compared to either vehicle or the uninjected, contralateral TA (Fig. 4a) without altering body weight appreciably (see Supplementary Fig. S7). The physiological cross-sectional area of ACE-083-injected muscles was increased by nearly 70% compared to uninjected, contralateral TA (Fig. 4b). In cross sections stained with hematoxylin and eosin, muscle fibers from ACE-083-injected muscle were visibly larger in diameter than those of the uninjected, contralateral muscle (Fig. 4c). Quantitative analysis of fiber diameter revealed a marked increase in the frequency of large-diameter fibers in the TA muscle as a function of ACE-083 treatment (Fig. 4d). For example, 55% of fibers from ACE-083-injected TA muscles were at least 51 μm in diameter, whereas only 6% of fibers from contralateral, uninjected TA muscles reached that threshold (Fig. 3D). These data indicate that local administration of ACE-083 in CMT mice causes focal muscle growth due to fiber hypertrophy.

We then investigated whether the muscle hypertrophic effect of local ACE-083 administration in CMT mice translates into increased force generation and improved ankle dorsiflexion. CMT mice were given unilateral injections of ACE-083 (100 µg) in the TA muscle twice weekly for 4 weeks. ACE-083 treatment increased basal isometric twitch force by 44% (Fig. 4e) and peak isometric tetanic force by 65% (Fig. 4f) in the injected muscle compared to the uninjected, contralateral muscle. As in wild-type mice, these ACE-083-mediated increases in absolute force did not translate into increased specific force (see Supplementary Fig. S6). However, ACE-083 treatment increased ankle dorsiflexion torque in the treated limb by 137%, as determined *in vivo* under isometric conditions, compared to that in vehicle-treated CMT mice (Fig. 4g). Finally, ACE-083 treatment in CMT mice reduced intramuscular levels of *Fbxo32* mRNA (encoding atrogin-1/MAFbx), an ubiquitin ligase that mediates muscle atrophy^36^, compared to the contralateral TA muscle (Fig. 4h). Together, these results indicate that local administration of ACE-083 in CMT mice causes focal muscle hypertrophy accompanied by increased generation of absolute force, increased ankle dorsiflexion torque and a beneficial change in a major biomarker of muscle atrophy.

### Focal skeletal muscle activity of ACE-083 in a mouse model of Duchenne muscular dystrophy

**Figure 5:**
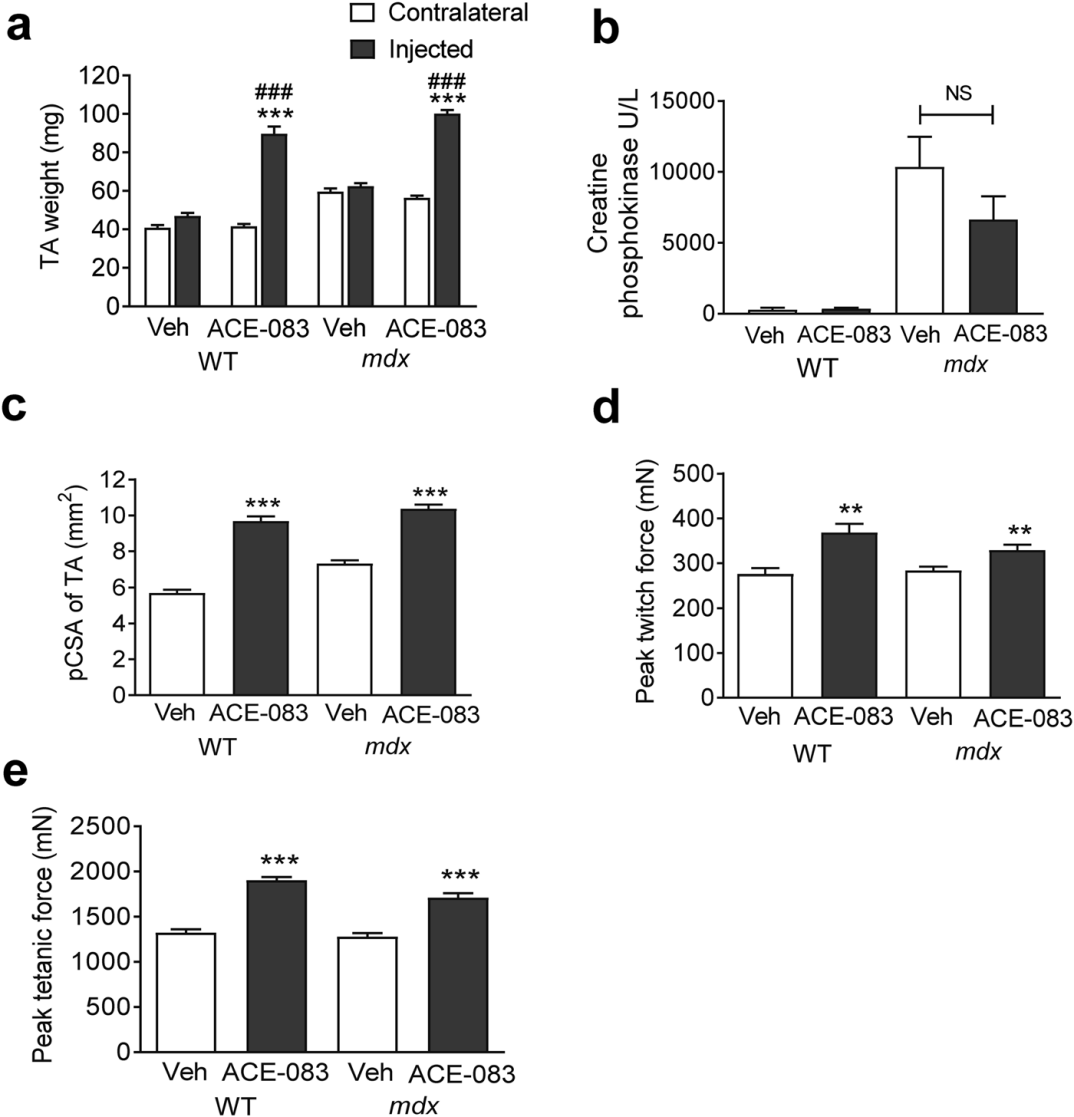
Local administration of ACE-083 in an *mdx* mouse model of Duchenne muscular dystrophy causes skeletal muscle growth and increased force generation. In these experiments, *mdx* and wild-type mice were given unilateral injections of ACE-083 (100 µg) or vehicle (PBS) in the TA muscle twice weekly for 4 weeks. (**a**) Weight of ACE-083-injected, vehicle-injected, and uninjected (contralateral) TA muscles in wild-type and *mdx* mice. Data are means ± SEM (n = 8–10). ***P ≤ 0.001 vs. uninjected muscle. ^###^P ≤ 0.001 vs. vehicle-injected muscle by one-way ANOVA with Tukey’s adjustment. (**b**) Serum concentrations of creatine phosphokinase in wild-type and *mdx* mice treated i.m. with ACE-083 or vehicle. Data are means ± SEM (n = 5 for vehicle-treated wild-type; n = 9–10 for other groups). **(c**) Physiological cross-sectional area (pCSA) of ACE-083-injected and vehicle-injected TA muscles in wild-type and *mdx* mice. Data are means ± SEM (n = 8–10). ***P ≤ 0.001 vs. vehicle-injected muscle by unpaired t-test. (**d**) Peak twitch force generated by ACE-083-injected and vehicle-injected TA muscles in wild-type and *mdx* mice as determined *in situ*. Data are means ± SEM (n = 8–10). **P ≤ 0.01 vs. vehicle-injected muscle by unpaired t-test. (**e**) Peak tetanic force generated by ACE-083-injected and vehicle-injected muscles in wild-type and *mdx* mice as determined *in situ*. Data are means ± SEM. (n = 8–10). ***P ≤ 0.001 vs. vehicle-injected muscle by unpaired t-test.

We lastly investigated effects of local ACE-083 administration on focal muscle growth, tissue integrity and corresponding muscle strength in an *mdx* mouse model of Duchenne muscular dystrophy. Four-week-old C57BL/10ScSn-Dmdmdx/J mice (*mdx* mice) and age-matched C57BL/10J wild-type control mice (Jax #000665) were given unilateral injections of ACE-083 (100 µg) or vehicle (PBS) in the TA muscle twice weekly for 4 weeks. ACE-083 treatment increased TA muscle weight by 77% in *mdx* mice and 116% in wild-type controls compared to the uninjected, contralateral TA (Fig. 5a) without altering body weight appreciably (see Supplementary Fig. S7). To determine whether ACE-083 improves muscle fiber integrity in this mouse model, we measured circulating concentrations of creatine phosphokinase, a biomarker of fiber damage known to be elevated in *mdx* mice^37^. As anticipated, *mdx* mice displayed markedly increased concentrations of creatine phosphokinase compared to wild-type mice (Fig. 5b). Although ACE-083 treatment reduced creatine phosphokinase concentrations in *mdx* mice by 36% (Fig. 5b), this change did not reach statistical significance, perhaps due to the focal nature of ACE-083 treatment effects. The physiological cross-sectional area of ACE-083-injected TA muscle was increased by 42% in *mdx* mice and by 70% in wild-type mice compared to vehicle-injected TA muscle in these respective mouse strains (Fig. 5c). ACE-083 treatment significantly increased peak twitch force of the TA muscle by 16% in *mdx* mice and 34% in wild-type mice compared to vehicle-treated muscle (Fig. 5d). Similarly, ACE-083 treatment significantly increased peak tetanic force of TA muscles by 34% in *mdx* mice and 44% in wild-type mice compared to vehicle-treated muscles (Fig. 5e). Thus, the magnitude of ACE-083 effects on isometric force generation in *mdx* mice were modest compared to those on muscle weight and cross-sectional area in this same model and smaller than its effects on force generation in CMT mice. As in the other models, ACE-083-mediated increases in absolute force did not translate into increased specific force (see Supplementary Fig. S6). Overall, these results indicate that intramuscular administration of ACE-083 in an *mdx* mouse model of muscular dystrophy causes focal growth of the injected muscle and increased generation of absolute force compared with vehicle treatment.

## Discussion

Individual skeletal muscles display remarkable diversity, leading to important clinical consequences. Besides differences in their developmental origin, biochemistry, morphology, and function, individual muscles differ in their susceptibility to heritable diseases, acquired diseases, and injury. For example, even muscle fibers that are histologically identical can exhibit dissimilar responses to disease and injury depending on their developmental origin^38^. Transcriptional profiling has revealed striking diversity of gene expression among skeletal muscles^1^, which not only underlies functional specialization of individual muscles but also contributes to their differential vulnerability to disease and potentially their responsiveness to therapy. Thus, there are inherent advantages in being able to treat individual muscles in a focused manner.

In the present study, we evaluated the ability of the follistatin-based fusion protein ACE-083 to produce localized muscle hypertrophy and increased strength in normal mice and murine models of neuromuscular disease. ACE-083 utilizes properties of FST291, an engineered, truncated variant of FST315 in which the intrinsic HBS is fully functional. For the ACE-083 construct, FST291 was linked to an IgG2 Fc domain for multiple purposes. Most importantly, we sought to confer the fusion protein with pharmacokinetic properties similar to those of an IgG molecule to extend the half-life of the protein in the pericellular microenvironment^27,39^. Moreover, attachment of an Fc domain doubles the number of HBS per protein molecule due to dimerization of FST291. An HBS slows the movement of proteins like follistatin within tissues and also enhances the clearance and degradation of extracellular proteins, including ligands bound to follistatin^41–43^. Finally, incorporation of an Fc domain assists in purification during protein production, and use of the IgG2 Fc isotype reduces the possibility of effector function due to immune cell engagement^40^. We have confirmed that ACE-083 in the general circulation is rapidly cleaved to inactive fragments in a manner similar to the related fusion protein FST288-Fc^23^, likely due to tissue-associated proteinases. Together, the inherent pharmacokinetic properties of the Fc domain, the paired HBS, and rapid proteolytic cleavage in the circulation promote localized action of ACE-083 in injected muscles.

Our results demonstrate that ACE-083 produces localized hypertrophy of skeletal muscle and improved muscle function in normal mice. We had previously found that intramuscularly administered FST288-Fc produces robust, dose-dependent growth of the injected gastrocnemius muscle in normal mice with no effects detected in other muscles^23^. Here we confirm that intramuscular administration of ACE-083 exerts a similar localized effect on the gastrocnemius muscle but have extended that finding in the context of a different skeletal muscle – the tibialis anterior – to show the growth-promoting effects of ACE-083 in normal mice are due to muscle fiber hypertrophy. This focal hypertrophic effect is accompanied by increased force generation during isometric contraction, consistent with previous studies of systemically acting interventions based on inhibition of Smad2/3 pathway signaling^17^.

Most importantly, we demonstrate here that ACE-083 exerts similar beneficial effects on muscle mass and function in a mouse model of CMT. This heterogeneous disease is the most common inherited neuropathy and one of the most frequent inherited diseases in humans, causing localized weakness and muscle atrophy that usually affects the legs and feet at onset and slowly progresses from distal to proximal musculature^2^. CMT is broadly classified as a demyelinating or axonal disease. TrJ mice carry the same L16P substitution in PMP22 found in patients with CMT1A^35^, the most common form of CMT. Like patients with CMT1A, TrJ mice exhibit weakness of the TA muscle, resulting in characteristic foot drop and locomotor impairment^44^. In this CMT model, intramuscular treatment with ACE-083 caused a robust increase in mass of the injected TA muscle due to myofiber hypertrophy, which translated into markedly increased force of isometric contraction and more than a doubling of dorsiflexion torque measured at the ankle. It is intriguing that these effects were accompanied in the injected muscle by a marked reduction in expression of *Fbxo32* mRNA encoding atrogin-1. Atrogin-1 is one of two ubiquitin ligases that serve as key mediators of muscle atrophy triggered by Smad2/3 signaling, among other transcription factors, in response to a wide range of stressors, including neural inactivity, muscle disuse, oxidative stress, and glucocorticoids^45^. This finding suggests one mechanism by which ACE-083 may produce beneficial effects on muscle function in neuropathic conditions.

We also evaluated ACE-083 efficacy in dystrophic muscle. In some dystrophies, such as FSHD, muscle degeneration and weakness is asymmetrical. The underlying defect in FSHD is aberrant expression of the double homeobox protein 4, *DUX4*^46^. This gene is not naturally expressed in mice, and attempts to model DUX4 myopathy in mice have until recently resulted in either excessive severity of disease or an inappropriate muscle phenotype^22,47^. Although the *mdx* mouse model of Duchenne and Becker muscular dystrophies used in this study does not exhibit asymmetric muscle wasting typical in FSHD, young *mdx* mice do exhibit active muscle necrosis, cellular infiltration, and myofibers with abnormalities typical of muscles in FSHD patients^47^. In the present study, intramuscular treatment with ACE-083 in young *mdx* mice caused a robust increase in mass of the injected TA muscle, which was accompanied by substantially increased force of isometric contraction. However, ACE-083-mediated increases in absolute force did not translate into increased specific force. This phenomenon of muscle strength increasing proportionally to muscle fiber size has been observed previously with systemic follistatin treatment and with other, systemic approaches to myostatin inhibition^18,21,48^. Focal treatment with ACE-083 also diminished the elevated serum levels of the enzymatic biomarker creatine phosphokinase in *mdx* mice, albeit not significantly, which is intriguing nonetheless because ACE-083 treatment was limited to a single muscle.

The robust activity of ACE-083 observed here depends on its ability to sequester and neutralize multiple TGFβ superfamily ligands acting as homeostatic inhibitors of muscle growth. Foremost among these ligands are activins, which are implicated as particularly important muscle regulators in primates^9^, and myostatin, which may play a more prominent role in rodents^8^. Interspecies differences in the relative involvement of these ligands could partly explain why inhibitory agents targeting myostatin alone have typically displayed greater efficacy in preclinical than clinical studies^49^. There are conflicting reports concerning a potential role for GDF11 signaling in muscle homeostasis, although this ligand can cause atrophy and inhibit regeneration of skeletal muscle *in vivo*^10–12^. Strong hypertrophic effects of follistatin on skeletal muscle have been observed in studies of muscle regulation by TGFβ superfamily ligands in mice with follistatin haploinsufficiency or overexpression^6,7,16,17^. In a direct comparison of naturally-occurring myostatin inhibitors administered to mice by systemic gene transfer, FST315 produced markedly greater increases in muscle mass and strength than other inhibitors^17^. Indeed, follistatin-based fusion proteins generate a focal muscle growth response after intramuscular administration comparable in magnitude to that of fusion proteins incorporating activin receptor type IIB (ActRIIB-Fc)^23^, which is consistent with their overlapping ligand-binding profiles. Previous studies have identified several BMPs that bind follistatin under certain conditions^29,30^. These BMPs are likely displaced from follistatin in the presence of higher-affinity ligands, particularly activins, as occurs with cognate type II receptors^50^. Here, we confirmed that ACE-083 binds BMP6 and BMP7 with intermediate affinity under cell-free conditions but does not interact with other BMPs. The absence of BMP9 interactions with ACE-083 differentiates this agent favorably from ActRIIB-Fc in terms of potential for unwanted vascular effects in non-muscle tissue^51^.

Skeletal muscle homeostasis in adulthood depends on a balance between opposing actions of two intracellular pathways that mediate canonical signaling by TGFβ superfamily ligands. Through binding to specific combinations of superfamily receptors, ligands such as activins, myostatin, and GDF11 trigger activation of Smad2/3 signaling, which coordinates changes in gene expression to stimulate protein degradation and muscle atrophy^4^, regardless of fiber type^52^. Through a similar mechanism, BMPs and other GDFs trigger activation of Smad1/5/8 signaling, which promotes protein synthesis, myofiber hypertrophy, and increased muscle mass^4^. Complex crosstalk between these two pathways occurs at multiple levels, providing a mechanistic basis for their functional antagonism in muscle homeostasis^4^. Although it can bind some BMPs, follistatin preferentially inhibits Smad2/3-pathway ligands, leading to activation of the Akt/mTOR pathway and increased protein synthesis in myofibers^33,53^. It is likely that some actions of endogenous follistatin are mediated by indirect activation of BMP-mediated Smad1/5/8 signaling.

Hence, an advantage of follistatin-based therapeutic interventions to promote muscle function is that they exploit the prominent role of endogenous follistatin, and Smad signaling generally, in orchestrating muscle adaptations to changing environmental demands. Importantly, *Fst* mutant mice exhibit haploinsufficiency, with *Fst*^+/−^ heterozygotes displaying significant reduction in muscle mass and impaired muscle function as well as impaired muscle regeneration after injury^7^. These findings indicate that endogenous follistatin exerts an important regulatory influence on Smad2/3 pathway signaling in adult muscle under both normal and pathologic conditions.

Skeletal muscle homeostasis is critically dependent on interactions between myofibers and multiple types of non-myofiber cells. Cellular interactions mediated by follistatin and its cognate ligands in this muscle microenvironment during muscle hypertrophy, atrophy, regeneration, or fibrosis are coming into sharper focus. In addition to mature myofibers, this niche consists of satellite (stem) cells and their activated progeny (myoblasts), interstitial cells such as fibroadipogenic progenitors, blood vessel-associated cells (pericytes), motor neurons, and immune cells such as macrophages^54–56^. The importance of this microenvironment is illustrated by its ability to restore functionality to satellite cells removed from dystrophic muscle^57^. In normal resting muscle, satellite cells express factors inhibitory to myogenesis, including myostatin and activin A, whereas fibroadipogenic progenitor cells express promyogenic factors such as follistatin^58^. Follistatin is also induced in myoblasts as they develop from satellite cells^59^. Most human satellite cells are reported to contain myostatin under basal conditions, while the number of myostatin-positive satellite cells in muscle fibers decreases after brief exercise^60^. Although the role of satellite cells in muscle hypertrophy remains controversial^60^, a decline in satellite cells has a major impact on myogenic competence, and inhibition of ActRIIB-pathway signaling with ActRIIB-Fc restores the regenerative potential of a reduced satellite cell population^58^.

These findings suggest that myogenesis and fibro/adipogenesis are tightly regulated by a balance between pro- and anti-myogenic signaling in the muscle niche. The functions of distinct populations of interstitial stem or progenitor cells in skeletal muscle are not well understood, but under pathologic conditions they can differentiate into fibrogenic, adipogenic, or other cell types^61–64^. It is therefore significant that Smad2/3-pathway activation by ligands such as myostatin not only regulates myoblast function but also induces fibroblast proliferation, reduces fibroblast apoptosis, and promotes fibrosis – which is reversible by ActRIIB-Fc – in skeletal muscle of mice^65,66^. Muscle-derived activin A inhibits muscle recovery from injury and contributes substantially to muscle degeneration since neutralization of activin A can promote muscle repair^67^. As noted previously^23^, follistatin overexpression improves tissue repair after muscle injury, and follistatin-based therapeutic interventions have shown signs of beneficial regenerative activity in preclinical models of muscular dystrophy or in preliminary clinical studies^17,19–22,68^.

It is important to distinguish circulating pools of endogenous follistatin and its cognate ligands from those in the muscle microenvironment. The differential ability of FST315 (circulating) and FST288 (locally acting) to bind heparan sulfate-containing proteoglycans in the pericellular space is considered to be a key determinant of their different biological activities, including the ability of FST288 to neutralize endogenous activin^15,41^. Extracellular proteoglycans act as molecular platforms on which TGFβ superfamily ligands in latent form are concentrated for local activation and/or release^69^. Thus, the high affinity of activin A prodomain for heparan sulfate concentrates prodomain-bound activin A in the vicinity of target cells. Similarly, circulating levels of myostatin do not reflect the extent of myostatin signaling in muscle tissue since most myostatin in this tissue is tethered extracellularly as inactive ligand (pro-myostatin) still covalently attached to its prodomain^70^. Therefore, concentration of locally-administered ACE-083 in the vicinity of these latent ligands is likely important for optimal ligand neutralization and robust muscle hypertrophy.

Our findings complement those of an earlier study^23^ and collectively provide a strong rationale for evaluating follistatin-based interventions such as ACE-083 in disorders with focal muscle weakness. ACE-083 promotes focal muscle growth in healthy volunteers^24^ and is being evaluated in phase 2 studies as a therapeutic agent for patients with CMT and FSHD^25,26^.

## Methods

### Construction, expression and purification of recombinant ACE-083 and FST315-Fc

Human FST315 was cloned by PCR from a commercially available nucleotide sequence encoding its 344-amino acid precursor (NCBI Reference Sequence: NP_037541.1) and ligated upstream of, and in-frame with, a sequence encoding human IgG1 Fc in the vector pAIDT.hFc. Human FST291 was cloned by PCR using FST315.hFc as template and ligated in the vector pAID4.hFc upstream of, and in-frame with, a sequence encoding human IgG2 Fc. This Fc sequence had been generated previously by PCR from a commercially available sequence and mutated from Asn to Lys at the 404 position (Kabat) for consistency with the most prevalent isotype allele. Plasmids were stably transfected in Chinese hamster ovary (CHO) DUKX cells and used to express ACE-083 (FST291-Fc) and FST315-Fc. Both proteins were purified according to a three-step procedure. The first purification step was performed as described^23^. The glycine eluate was neutralized before being loaded over a Q Sepharose Fast Flow anion-exchange column (GE Healthcare, Boston, MA), followed by wash and elution steps with an intermediate NaCl concentration. For ACE-083, the Q eluate was then loaded onto a SP Sepharose cation-exchange column (GE Healthcare, Boston, MA), then washed and eluted with an intermediate NaCl concentration. The final material was dialyzed into a pH 6.5 citrate buffer. For FST315-Fc, the SP Sepharose step was replaced by a preparative size-exclusion column, and the final material was dialyzed in phosphate-buffered saline. Characterization of the final products was performed as described^23^.

### Affinity of fusion proteins for heparin

ACE-083 and FST315-Fc (200 μg) were injected separately onto a 7.5 mm × 7.5 cm TSKgel Heparin-5PW column (Tosoh) equilibrated with 50 mM Tris and 150 mM sodium chloride (pH 8.0). Protein was eluted using a linear gradient of 0–100% 50 mM Tris and 2 M sodium chloride (pH 8.0). Relative affinity of each protein for immobilized heparin was based on the concentration of sodium chloride necessary to elute bound protein from the column.

### Affinity of fusion proteins for extracellular matrix extract

Corning BioCoat Matrigel Assay Plates (Corning, NY) were blocked with 200 μL/well 100% Pierce StartingBlock (VWR, Radnor, PA). ACE-083 and FST315-Fc were serially diluted in blocking buffer (0–60 nM) and applied to the plate in triplicate (200 μl). The plates were incubated for 2 hours at room temperature, then washed four times with TBS containing 0.1% Tween 20 and 10% StartingBlock. This was followed by a 2-hour incubation with HRP-goat-anti-human IgG-Fc (1:5000; Jackson Immunoresearch, West Grove, PA). The plates were washed four times and then TMB substrate (100 μl; KPL, Gaithersburg, MD) was added. Stop solution was used to halt the colorimetric reaction, which was read at a wavelength of 450 nm using a VersaMax microplate reader (Molecular Devices, San Jose, CA).

### Characterization of ligand binding to ACE-083

Ligand binding affinities for ACE-083 were determined by surface plasmon resonance (SPR) using a Biacore T100 instrument (GE Healthcare). Kinetic assays were performed in a capture format at 37 °C as described^23^, with the following changes: a concentration series of activin A (0.156 nM–10 nM), activin B (0.0098 nM–2.5 nM), GDF8 (0.156 nM–40 nM) and GDF11 (0.078 nM–10 nM) were injected over captured ACE-083 in duplicate with an association time of 210 sec and a dissociation time of 600 sec (1200 sec for activin A and GDF11). Methodology for determining BMP binding to ACE-083 by surface plasmon resonance can be found in Supplementary Methods.

### Cell-based reporter-gene assay

A reporter-gene assay was used to evaluate the effects of ACE-083 on signaling by TGFβ superfamily ligands. The CAGA12 motif is present in the TGFβ-responsive gene PAI-1 so this vector is of general use for factors signaling through Smad2 and Smad3. A204 cells were co-transfected with a pGL3 CAGA12-luciferase reporter plasmid^71^ and a control Renilla reporter plasmid (pRLCMV-luciferase) using OptiMEM media (Invitrogen, Carlsbad, CA). After 24 hours, the assay was conducted with activin A, activin B, GDF8, and GDF11 as described^23^, except with ACE-083 as the test agent. IC_50_ values were calculated using GraphPad Prism.

### Animal studies

Experimental procedures were performed according to protocols approved by the Acceleron Pharma Institutional Animal Care and Use Committee. All studies were performed in accordance with the relevant guidelines and regulations. Wild-type C57BL/10SnJ (BL/10) mice, 4-week-old male C57BL/10ScSn-Dmdmdx/J (*mdx*) mice and 7-month old B6.D2-Pmp22Tr-J/J mice were obtained from Taconic (Germantown, NY). ACE-083 or vehicle (phosphate-buffered saline, PBS) were administered in an injection volume of 50 μl. Assessments of body weight and muscle force generation were made at intervals throughout the study. At the conclusion of the study, serum was collected, muscle weights obtained, and tissue processed for immunohistochemistry and PCR. In some experiments, serum levels of follicle-stimulating hormone and creatine phosphokinase were measured by immunoassays performed by Eurofins Pharma Bioanalytical Services US Inc. (St. Charles, MO) and IDEXX (Westbrook, ME), respectively.

### Taqman real-time PCR

Muscle tissues were analyzed as described^52^.

### Histological analysis of myofibers

TA muscles were snap frozen in 2-methylbutane (isopentane). Serial cross sections (8-µm thick) were cut using a cryostat microtome (Slee Pearson, UK). To visualize myofiber boundaries, muscle sections from *mdx* mice were stained with hematoxylin-eosin, and sections from CMT mice were immunostained with an antibody directed against laminin, a major component of the basal lamina. Digital photographs were taken and images analyzed as described^52^.

### Contractile properties of TA muscle during isometric contraction *in situ*

Mice were anesthetized with isoflurane (Fig. 4) or sodium pentobarbital (Figs. 3, 5; 80 mg/kg, i.p.). A small incision was made to reveal the distal tendons of the ankle dorsiflexors. A silk suture loop was securely tied to the TA tendon just distal to the myotendenous junction, and the tendons of the extensor digitorum longus, extensor hallucis longus, and TA (distal to the suture) were severed. The animal was placed supine on a platform maintained at 38 °C. The knee was immobilized using a 27 gauge needle inserted through the knee joint and clamped to the platform (Fig. 4) or by a silk suture passed beneath the patellar tendon used to securely fasten the knee against a stationary horizontal rod (Figs. 3, 5). The TA tendon suture was attached to a calibrated dual mode muscle lever system (model 305C-LR, Aurora Scientific). A biphasic muscle stimulator (model 701 A, Aurora Scientific) delivered supramaximal, 200 µs square-wave pulse to platinum needle electrodes inserted superficially onto the TA muscle (Fig. 4) or inserted behind the knee for stimulation of the peroneal nerve (Figs. 3, 5). Tetanic force was evaluated using 200 ms trains of supramaximal stimuli at 200 Hz. Optimal length was determined by systematically altering muscle length until tetanic force was maximized. The force-frequency relationship was determined by stimulating the muscle at frequencies from 10 to 200 Hz.

### Measurement of ankle dorsiflexion torque *in vivo*

Mice were anesthetized under isoflurane and placed on an aluminum platform maintained at 38°C. The knee was securely fastened to an immobile horizontal support. The foot was taped to the foot pad of a calibrated dual mode muscle lever system (model 305C-LR, Aurora Scientific). Platinum needle electrodes (model F-E2M-48, Grass Technologies) were inserted superficially onto the TA muscle for muscle stimulation via a biphasic muscle stimulator (model 701 A, Aurora Scientific). Twitch force was determined with a single stimulus consisting of a 200-µs square-wave pulse. The tetanic force-frequency relationship was generated with square-wave stimuli of 200 ms.

### Statistical analysis

Data are reported as means ± SE standard error of the mean (SEM) unless indicated otherwise. Data were analyzed by unpaired Student t-test, one-way analysis of variance (ANOVA) followed by either Tukey or Dunnett’s method or by Mann-Whitney *U* test. P ≤ 0.05 was considered statistically significant. GraphPad Prism version 7.0 (GraphPad Software, San Diego, CA) was used for statistical analysis.

## Supplementary information

Supplementary Information

## Data Availability

All data generated or analysed during this study are included in this published article or available from the corresponding author, Dr. Scott Pearsall, email spearsall@xlrn.com upon reasonable request.
