# Supplementary material for: Follistatin-based ligand trap ACE-083 induces localized hypertrophy of skeletal muscle with functional improvement in models of neuromuscular disease
Source: Sci Rep. 2019 Aug 6;9:11392. doi: 10.1038/s41598-019-47818-w (PMC6684588; doi:10.1038/s41598-019-47818-w)
Supplement: Supplementary file 1 — Supplementary Information [file 41598_2019_47818_MOESM1_ESM.pdf]

Supplementary Materials

Follistatin-based ligand trap ACE-083 induces localized hypertrophy of skeletal muscle with functional improvement in models of neuromuscular disease.

R. S. Pearsall, M. V. Davies, M. Cannell, J. Li, J. Widrick, A. W. Mulivor, S. Wallner, M. E. Troy, M. Spaits, K. Liharska, D. Sako, R. Castonguay, S. Keates, A. V. Grinberg, R. N. V. S. Suragani, R. Kumar.

Affiliation: Acceleron Pharma, Cambridge, Massachusetts 02139, USA

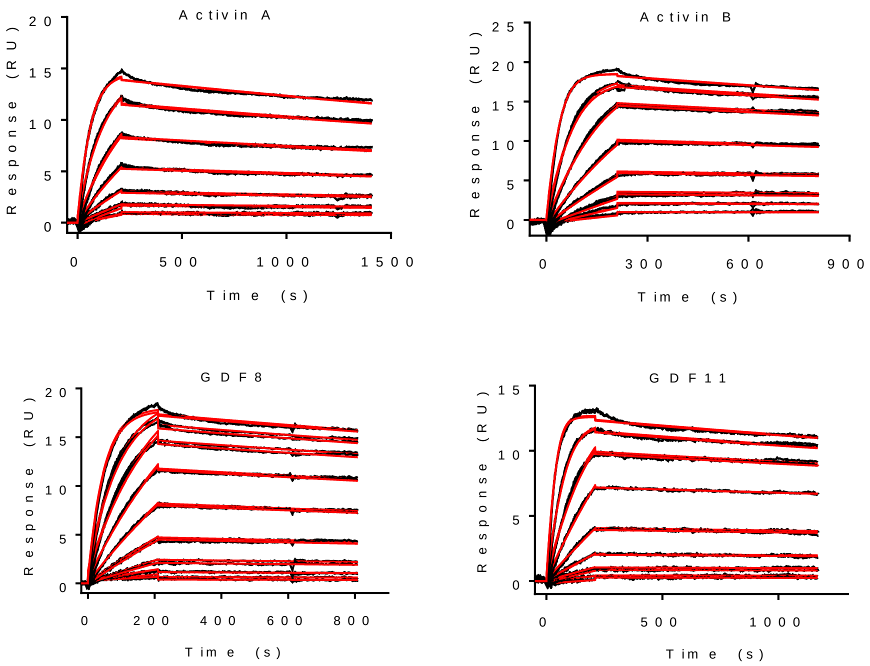

Supplementary Figure S1. Kinetic characterization of ACE-083 binding to TGF-β superfamily ligands activin A, activin B, myostatin (GDF8) and GDF11 by surface plasmon resonance. Purified ACE-083 was captured on an anti-human Fc IgG chip and ligands were injected over the captured receptor at 37 °C. SPR assays were carried out for ACE-083 more than three times, this data represents means of two experiments performed on separate flow cells. Sensograms (black lines) are overlaid with fits to a 1:1 interaction model with mass transport limitations (red lines). A summary of the kinetic data from these experiments is presented in Table 1.

Ligand	k_a ($M^{-1}s^{-1}$)	k_d (s^{-1})	K_D (pM)
BMP2	No binding		
BMP3	No binding		
BMP4	No binding		
BMP5	No binding		
BMP6	2.45×10^{6}	1.01×10^{-4}	422
BMP7	6.75×10^{6}	1.96×10^{-3}	291
BMP8a	No binding		
BMP9	No binding		
BMP10	No binding		
BMP13 (GDF6)	No binding		
BMP14 (GDF5)	No binding		
BMP15 (GDF9b)	No binding		

Supplementary Table 1. ACE-083 binding to BMP ligands as determined by surface plasmon resonance. Data for kinetic characterization were duplicates globally fit to a 1:1 binding model with mass transfer term using BIAevaluation software and are means of two separate experiments performed on separate flow cells.

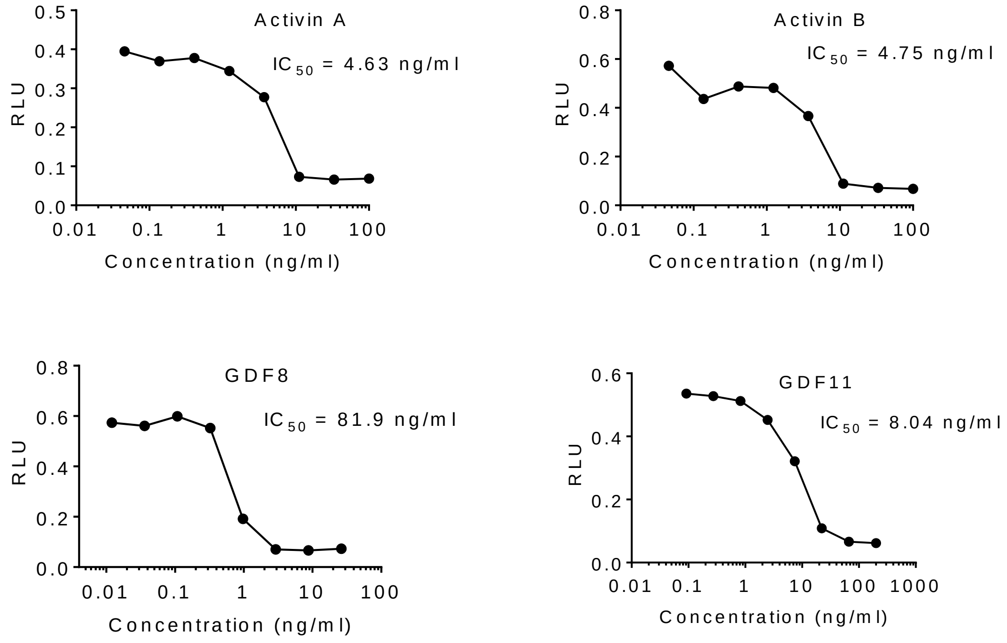

Supplementary Figure S2. ACE-083 potently inhibits activity of TGF-β superfamily ligands activin A, activin B, myostatin (GDF8) and GDF11 in a cell-based reporter gene assay. Luciferase activity in A204 cells was measured at each concentration of ACE-083. These graphs are representative of three independent experiments used to generate the values shown in Table 1. Experimental values were normalized to the control value and expressed as the ratio of relative luciferase units (RLU). The IC_{50} value for each curve was calculated using XLfit.

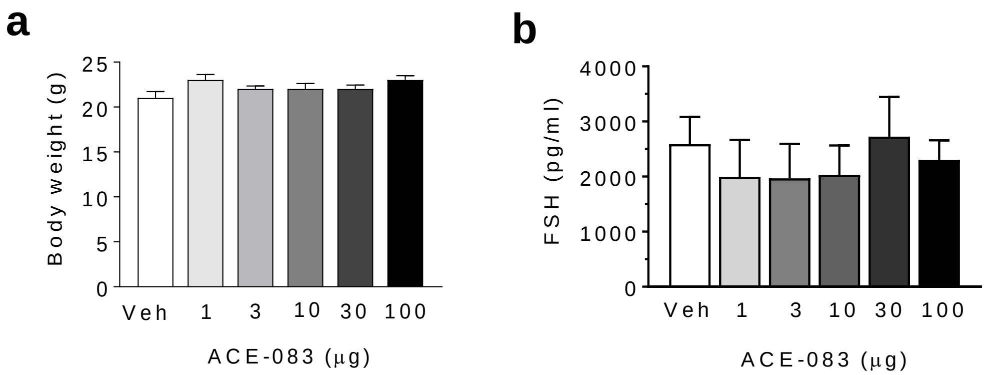

Supplementary Figure S3. Intramuscular administration of ACE-083 in wild-type mice alters neither body weight nor circulating concentrations of follicle-stimulating hormone (FSH). In these experiments, the gastrocnemius muscle was injected unilaterally with ACE-083 or vehicle twice weekly for 4 weeks. (a) Body weight as a function of ACE-083 dose. Data are means ± s.e. (n = 8). (b) Serum concentrations of FSH as a function of ACE-083 dose. Data are means ± s.e. (n = 4).

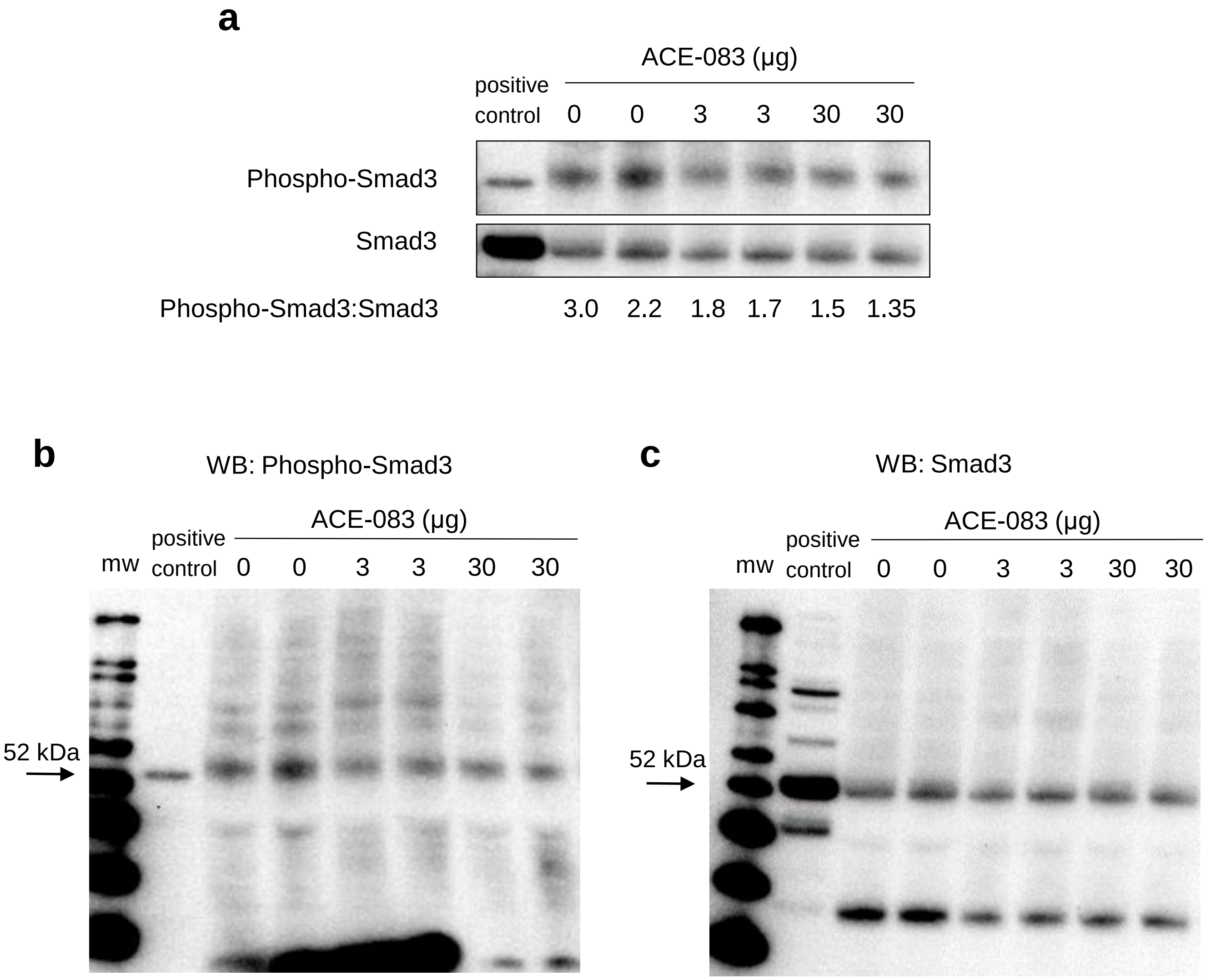

Supplementary Figure S4. **ACE-083 treatment decreases Smad3 phosphorylation in the TA muscle.** Wild type mice were injected unilaterally in the TA muscle with ACE-083 (3-30 μg) or vehicle (0 μg) twice weekly for 4 weeks. Muscle lysates were prepared and then subjected to Western blot analysis. (a) Cropped blots show levels of phosphorylated Smad3 (upper panel) and total Smad3 (lower panel). In the left lane of each cropped blot is a positive control for the protein of interest. The ratio of phospho-Smad3 to total Smad3 is shown for each lane. (b) Full-length Western blot for phospho-Smad3. (c) Full-length Western blot for total Smad3.

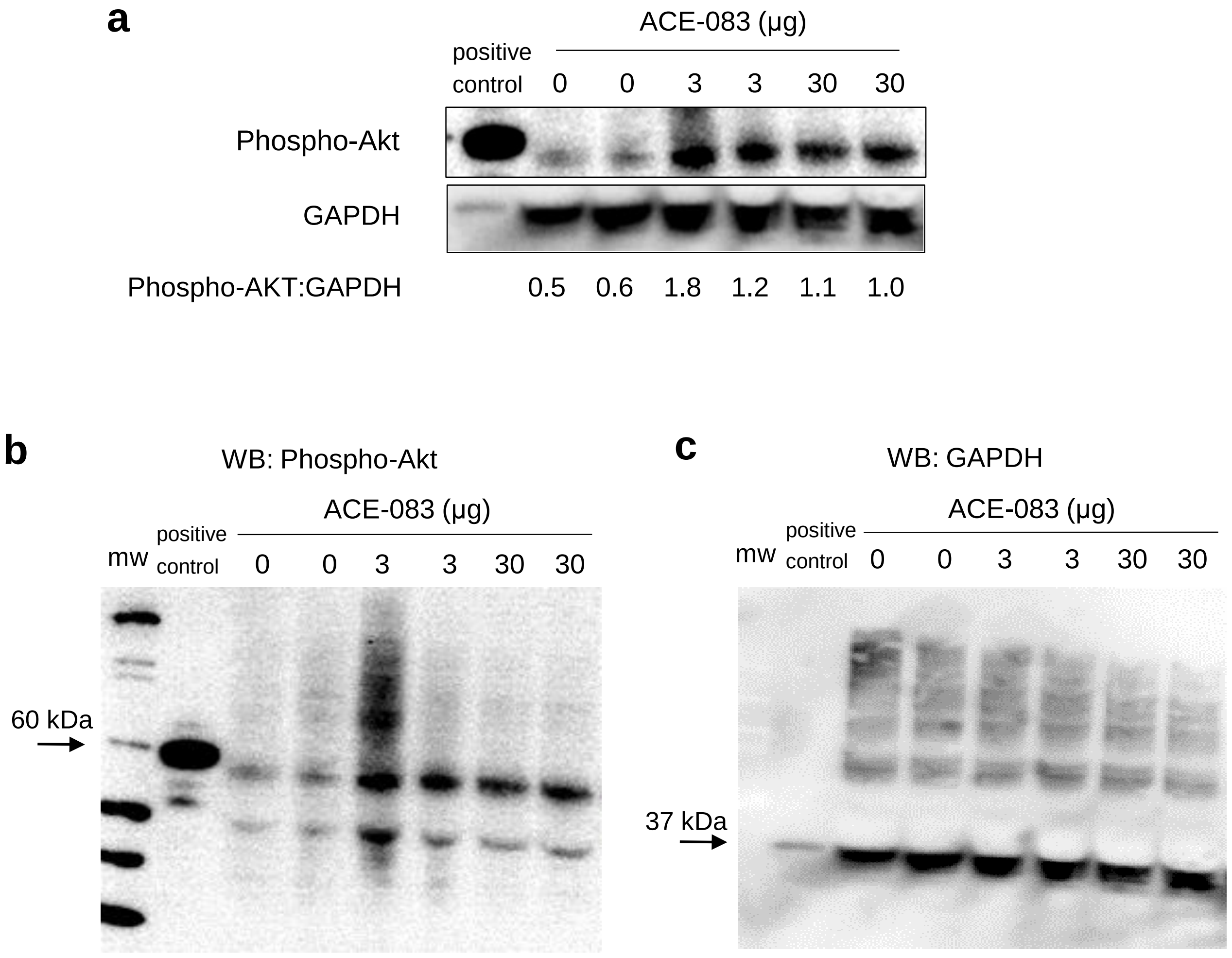

Supplementary Figure S5. **ACE-083 treatment increases Akt activation in the TA muscle.** Wild-type mice were injected unilaterally in the TA muscle with ACE-083 (3-30 μg) or vehicle (0 μg) twice weekly for 4 weeks. Muscle lysates were subjected to Western blot analysis. (a) Cropped blots show levels of phosphorylated Akt (upper panel) and loading control GAPDH (lower panel). On the left-hand side of each blot is a positive control for the protein of interest. The ratio of phosphorylated Akt to GAPDH is shown under each lane of the blot. (b) Full-length Western blot for phospho-Akt. (c) Full-length Western blot for GAPDH.

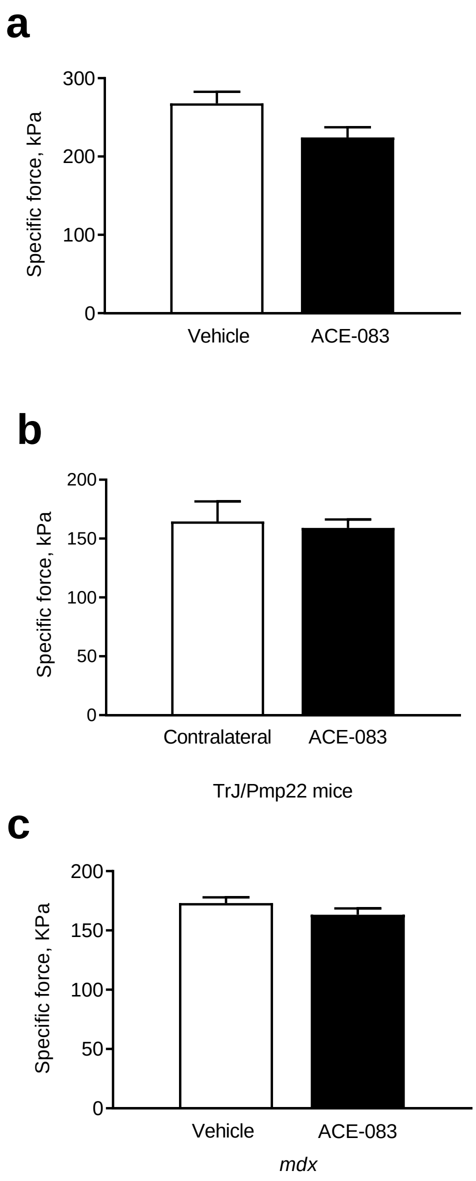

Supplementary Figure S6. Intramuscular administration of ACE-083 does not enhance specific force in wild type, TrJ/Pmp22 or *mdx* mice. (a) Specific force generated by ACE-083-injected and vehicle-injected TA muscles in wild type C57BL/6 mice. Data are means ± SEM (n = 6-8). (b) Specific force generated by ACE-083-injected and contralateral TA muscles in TrJ/Pmp22 mice. Data are means ± SEM. (n = 5). (c) Specific force generated by ACE-083-injected and vehicle-injected TA muscles in *mdx* mice. Data are means ± SEM. (n = 11).

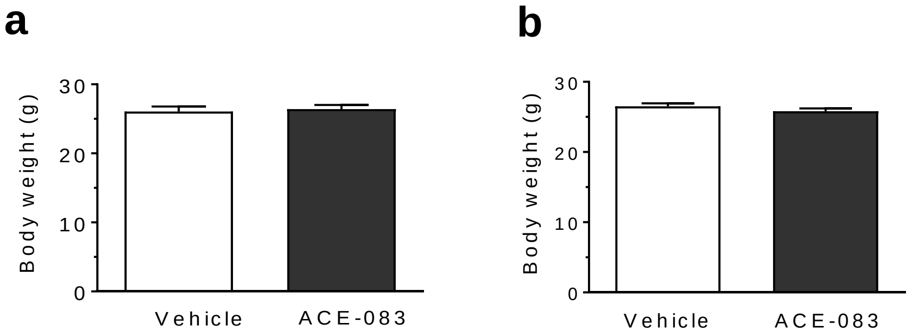

Supplementary Figure S7. Intramuscular administration of ACE-083 into TrJ/Pmp22 mice (CMT mice) or *mdx* mice does not affect body weight. Mice were injected unilaterally in the TA muscle with ACE-083 (100 µg) or vehicle twice weekly for 4 weeks. (a) Body weights of CMT mice treated i.m. with ACE-083 or vehicle. Data are means ± s.e. (n = 10-12). (b) Body weight of *mdx* mice treated i.m. with ACE-083 or vehicle. Data are means ± s.e. (n = 10).

Supplementary Methods

Characterization of BMP ligand binding to ACE-083 by surface plasmon resonance. As described in the manuscript, ACE-083 was captured on an experimental flow cell containing immobilized goat anti-human Fc-specific antibody. BMP2, BMP3, BMP4, BMP5, BMP6, BMP7, BMP8a, BMP9, BMP10, BMP13 (GDF6), BMP14 (GDF5) and BMP15 (GDF9b) (purchased from R&D Systems, except for BMP13, purchased from Peprotech, Rocky Hill, NJ) were injected at 10 and 100 nM with an association time of 210 sec and a dissociation time of 600 sec at a flow rate of 100 μl/min. The antibody surface was regenerated between binding cycles by injection of 10 mM glycine, pH 1.7. Buffer containing 0.01 M HEPES, 0.5 M NaCl, 3 mM EDTA, 0.005% v/v Surfactant P20, pH 7.4 supplemented with 0.5 mg/mL BSA was used as running buffer. All sensorgrams were processed by double referencing (subtraction of the responses from the reference surface and from an average of blank buffer injections).
From all the BMP ligands tested, BMP6 and BMP7 showed binding to ACE-083 at 10 and 100 nM. Detailed kinetic analysis was performed, where ACE-083 was captured on an experimental flow cell containing immobilized goat anti-human Fc-specific antibody. A concentration series of BMP6 (0.156 nM–5 nM) and BMP7 (0.078 nM–5 nM) were injected with two-fold serial dilution with an association time of 210 sec and a dissociation time of 600 sec at a flow rate of 100 μl/min in duplicate. The experiment was performed on two different flow cells and results shown are an average of both experiments. To obtain kinetic rate constants, the corrected data were fit to 1:1 interaction model which includes a term for mass transport using BIAevaluation software (GE Healthcare). The equilibrium dissociation constant K_D was determined by the ratio of binding rate constants k_a/k_d.

Western blotting. Lysates for Western blotting were prepared by homogenizing muscle tissue in cold RIPA buffer supplemented with protease inhibitors (1:100) and centrifuged to remove any unwanted tissue fragments. Total protein in muscle lysates was measured using an RC DC Protein Assay Kit II (BioRad). Approximately 18 μg of protein was loaded onto an SDS-PAGE gel then transferred to PVDF membrane. Western blotting was performed with primary antibodies to phospho SMAD3 (Ser423/425), SMAD3, phospho AKT (Ser473), GAPDH (Cell Signaling Technology, Danvers, MA). For detection, a HRP-conjugated goat anti-rabbit secondary antibody was used (R&D Systems, Minneapolis, MN). Bands were visualized using SuperSignal West Pico Chemiluminescent Substrate (Thermo Fisher Scientific, Waltham, MA).
